# Deoxyvasicinone with Anti-Melanogenic Activity from Marine-Derived *Streptomyces* sp. CNQ-617

**DOI:** 10.3390/md20020155

**Published:** 2022-02-21

**Authors:** Se-eun Lee, Min-ju Kim, Prima F. Hillman, Dong-Chan Oh, William Fenical, Sang-Jip Nam, Kyung-Min Lim

**Affiliations:** 1 The Graduate School of Industrial Pharmaceutical Sciences, Ewha Womans University, Seoul 03760, Korea; 0213lse@naver.com (S.-e.L.); bm4976@gmail.com (M.-j.K.); 2 Department of Chemistry and Nanoscience, Ewha Womans University, Seoul 03760, Korea; primafitriah@gmail.com; 3 Natural Products Research Institute, College of Pharmacy, Seoul National University, Seoul 08826, Korea; dongchanoh@snu.ac.kr; 4 Center for Marine Biotechnology and Biomedicine, Scripps Institution of Oceanography, University of California, San Diego, CA 92093-0204, USA; wfenical@ucsd.edu; 5 College of Pharmacy, Ewha Womans University, Seoul 03760, Korea

**Keywords:** *Streptomyces* sp., deoxyvasicinone, marine natural products, anti-melanogenic effect

## Abstract

The tricyclic quinazoline alkaloid deoxyvasicinone (**DOV**, **1**) was isolated from a marine-derived *Streptomyces* sp. CNQ-617, and its anti-melanogenic effects were investigated. Deoxyvasicinone was shown to decrease the melanin content of B16F10 and MNT-1 cells that have been stimulated by α-melanocyte-stimulating hormone (α-MSH). In addition, microscopic images of the cells showed that deoxyvasicinone attenuated melanocyte activation. Although, deoxyvasicinone did not directly inhibit tyrosinase (TYR) enzymatic activity, real-time PCR showed that it inhibited the mRNA expression of TYR, tyrosinase-related protein 1 (TRP-1), and tyrosinase-related protein 2 (TRP-2). In the artificial 3D pigmented skin model Melanoderm^TM^, deoxyvasicinone brightened the skin significantly, as confirmed by histological examination. In conclusion, this study demonstrated that the marine microbial natural product deoxyvascinone has an anti-melanogenic effect through downregulation of melanogenic enzymes.

## 1. Introduction

Melanin is a group of pigments that occur throughout the body of various animals. Melanin determines the skin tone of humans [1] and plays an important role in absorbing UV radiation and protecting the skin from oxidative damage [2]. Melanin production refers to synthesis of melanin from the melanin-producing cells, or melanocytes in the epidermis [3,4]. Melanin is a mixture of the dark brown or black eumelanin and the lighter-colored pheomelanin [5]. Melanin production is accomplished by many activators, especially tyrosinase (TYR), tyrosinase-related protein-1 (TRP-1), and tyrosinase-related protein-2 (TRP-2) [6]. Specifically, TYR catalyzes the initial process of melanin production, thus providing a common substrate for the synthesis of eumelanin and pheomelanin; TYR catalyzes the oxidation of *L*-tyrosine to *L*-dopaquinone (*L*-DQ) or *L*-dihydroxyphenylalanine (*L*-DOPA) and the oxidation of *L*-DOPA to *L*-DQ and the transformation of 5,6-dihydroxyindole (DHI) to indole-5,6-quinone [7]. Meanwhile, TRP-2 and TRP-1 catalyze the transformation of dopachrome to DHICA (indole-5,6-quinone carboxylic acid), and of DHICA to eumelanin [8]. As a result, melanogenesis is regulated by enzymatic activity and the expression levels of TYR, TRP-1, and TRP-2. The inhibition of enzymatic activity of TYR restricts melanocytes from initiating melanogenesis, and suppression of TRP-1 and TRP-2 restrains the synthesis of eumelanin [5]. Studies for novel mechanisms and new anti-melanogenic agents are ongoing to correct excessive melanin accumulation and cosmetic applications, and to correct medical problems arising from melanocyte activation [9].

Recently, marine-derived actinomycete bacteria have been identified as an important source of structurally unique secondary metabolites with a variety of biological activities such as antimicrobial, antiviral, and anti-tumor activity, as well as cytotoxicity [10,11,12]. Most of the known antimicrobials today were originally isolated from actinomycete, especially from the genus *Streptomyces*. The genus *Streptomyces* (Actinobacteria) is a rich source of secondary metabolites and provides more than 80% of commercially available antibiotics [13,14].

**Figure 1:**
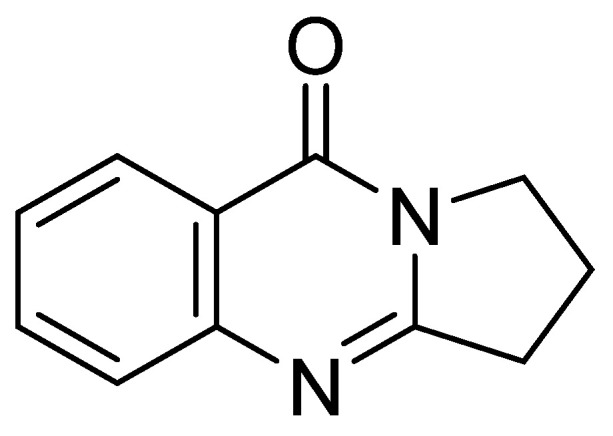
The structure of deoxyvasicinone (**1**).

For example, deoxyvasicinone was first isolated from a *Streptomyces* sp., displaying antimicrobial, anti-inflammatory, and anti-depressant activities [15]. However, the biological activity of this compound with respect to an anti-melanogenic effect has not been previously reported. HPLC-UV-guided fractionation of the culture broth of *Streptomyces* sp. CNQ-617, isolated from marine sediment collected from offshore of La Jolla, California, has led to the discovery of deoxyvasicinone (**1**) (Figure 1). Herein, we describe the isolation of deoxyvasicinone (**1**) from the marine microorganism, *Streptomyces* sp. CNQ-617, and demonstrate the anti-melanogenic effects of compound **1** in vitro using murine and human melanoma cells and in a human artificial pigment 3D skin model in an effort to develop a new functional cosmetic ingredient.

## 2. Results

### 2.1. Identification of Deoxyvasicinone

Compound **1** was isolated as a white solid, and its ion peak at *m*/*z* 187.5 [M+H]^+^ was revealed in the low resolution mass spectrum (LRMS). The ^1^H NMR spectrum (Figure S1) of **1** displayed 4 protons of 1,2-disubstituted aromatic ring at *δ*_H_ 8.20 (1H, d, *J* = 8.0 Hz), 7.81 (1H, dd, *J* = 8.0, 8.0 Hz), 7.60 (1H, d, *J* = 8.0 Hz), and 7.52 (1H, dd, *J* = 8.0, 8.0 Hz). Analysis of ^13^C nuclear magnetic resonance (NMR) (Figure S2) and heteronuclear single quantum coherence (HSQC) spectroscopic data of **1** revealed 11 carbon signals including three methylenes, four methines, and four fully-substituted carbons. On the basis of the interpretation of the MS and ^1^H NMR spectroscopic data, and by comparison of the NMR data to those of previous reports [16], we identified compound **1** as deoxyvasicinone.

### 2.2. The Effect of Deoxyvasicinone on Melanogenesis and Viability of Rodent of B16F10 Cells

**Figure 2:**
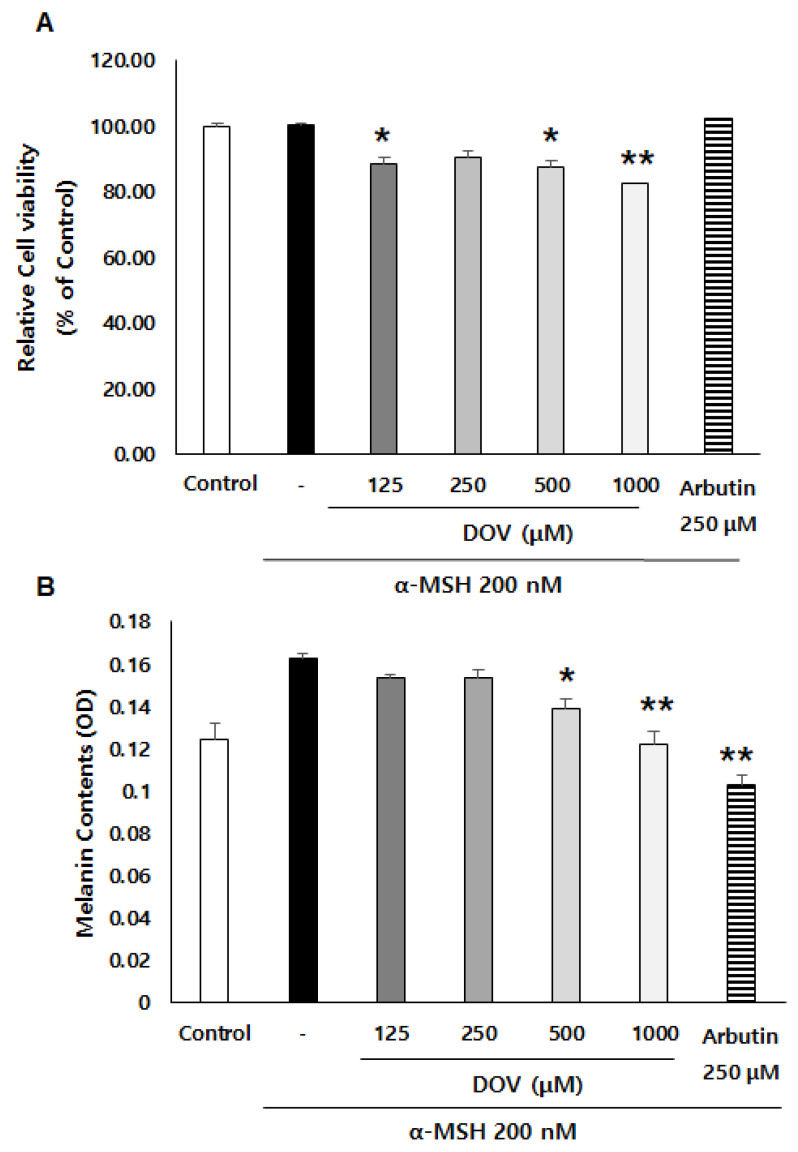

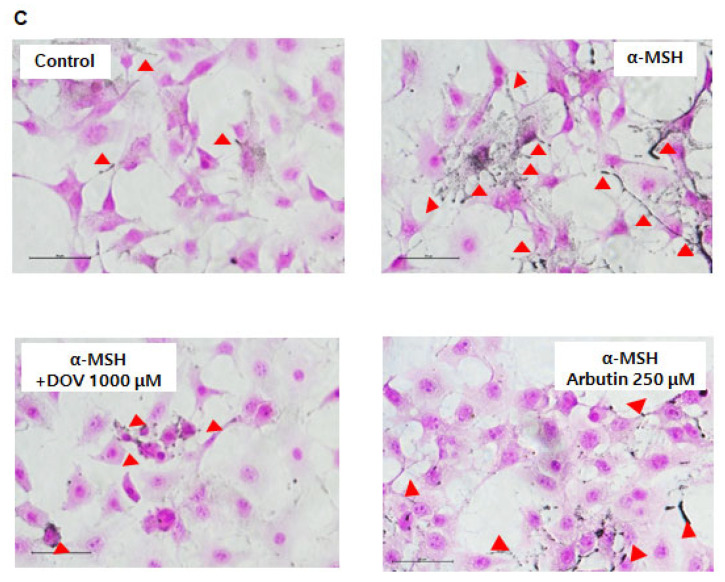
The effects of deoxyvasicinone (125–1000 μM) or 250 μM arbutin (a positive control) on the cell viability and melanin contents of α-MSH (200 nM)-stimulated B16F10 cells: (**A**) MTT assay (cell viability); (**B**) intracellular melanin content assay; (**C**) FM stain (melanin is stained dark black). Red arrows indicate dendrite formation from melanocytes. Scale bar is 50 μm. Values are presented as the mean ± SD (*n* = 3, * *p* < 0.05 and ** *p* < 0.01 by Student’s *t*-test). Control—cells treated with neither α-MSH nor chemicals, DOV—deoxyvasicinone.

The anti-melanogenic effects of deoxyvasicinone were investigated on α-MSH-stimulated B16F10 murine melanoma cells, as described previously [16]. After treatment of the cells with various concentrations of deoxyvasicinone (125–1000 μM) or 250 μM arbutin as a positive control along with α-MSH (200 nM) for 48 h, the melanin contents and cell viability were measured and compared with the untreated control group (Control) or the α-MSH alone group, as presented in Figure 2A. Deoxyvasicinone slightly reduced cell viability (up to 80% of untreated control at 1000 μM). Meanwhile, as shown in Figure 2B, the treatment of deoxyvasicinone attenuated the α-MSH-stimulated intracellular melanin increase in the B16F10 cells in a concentration-dependent manner. Furthermore, the microscopic observation of B16F10 cells stained with Fontana–Masson (FM), which stains melanin dark black, confirmed that melanin synthesis and dendrite formation stimulated by α-MSH were inhibited by the treatment with 1000 μM deoxyvasicinone or 250 μM arbutin, as a positive control (Figure 2C).

### 2.3. The Anti-Melanogenic Effects of Deoxyvasicinone upon MNT-1 Cells

**Figure 3:**
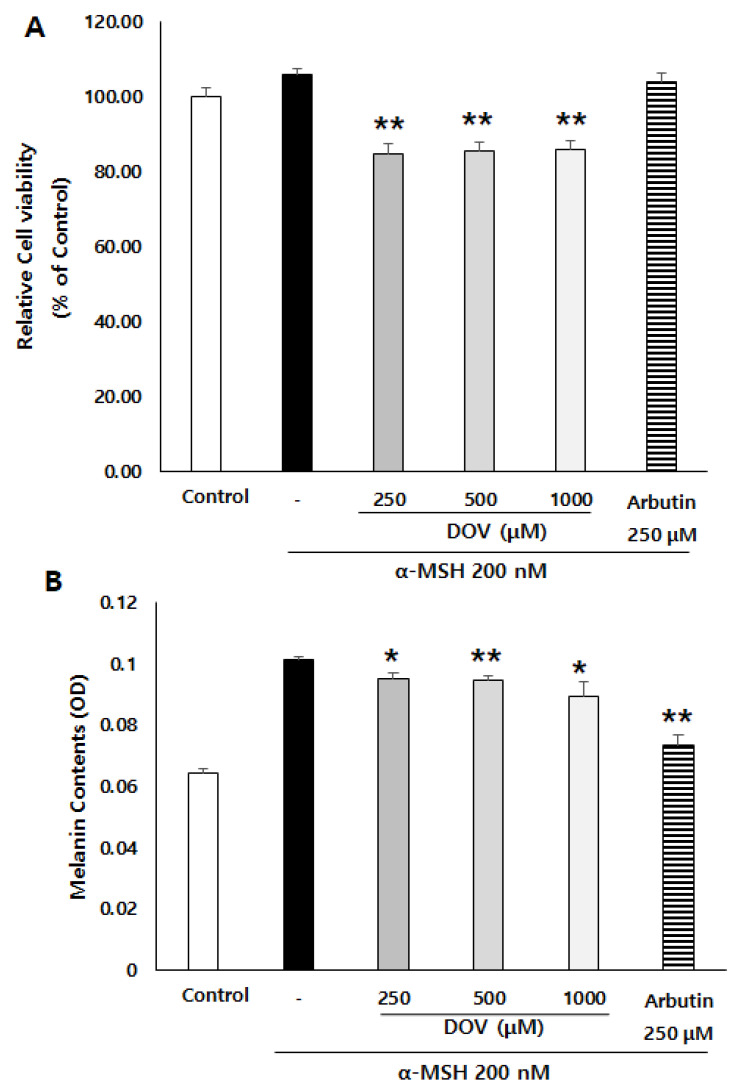
The effects of deoxyvasicinone (250–1000 μM) or arbutin (a positive control) on the melanin content and viability of α-MSH-stimulated human melanoma cells, MNT-1. (**A**) Cell viability as determined with MTT assay. (**B**) Intracellular melanin assay. Values are presented as the mean ± SD (*n* = 3, * *p* < 0.05 and ** *p* < 0.01 by Student’s *t*-test). Control–cells treated with neither α-MSH nor chemicals.

To confirm whether deoxyvasicinone could also have anti-melanogenic effects in human melanocytes, we employed MNT-1 cells, a human melanoma cell line. α-MSH-stimulated MNT-1 cells were treated with 250–1000 μM deoxyvasicinone for 48 h, after which the cell viability and the intracellular melanin contents were measured. As shown in Figure 3A,B, deoxyvasicinone treatment decreased the melanin contents of the MNT-1 cells while cell viabilities were maintained above 80% of the untreated control, confirming that deoxyvasicinone has anti-melanogenic effects in both human and murine melanocytes.

### 2.4. Effects of Deoxyvasicinone Treatment upon MNT-1 and HaCaT Co-Cultured Cells

**Figure 4:**
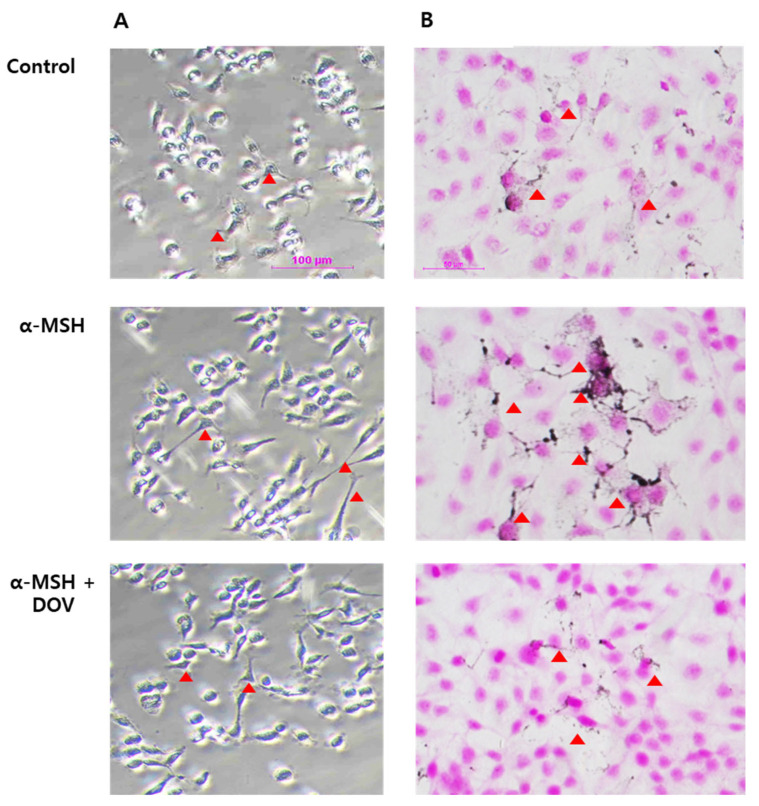
The microscopic images of α-MSH-stimulated MNT-1/HaCaT co-culture treated with deoxyvasicinone (1000 μM). (**A**) MNT-1/HaCaT co-culture stained by *L*-DOPA (scale bar = 100 μm); (**B**) MNT-1/HaCaT co-culture stained by FM staining (scale bar = 50 μm; arrow heads indicated dendrite formation). Control—cells treated with neither α-MSH nor chemicals; α-MSH (200 nM); DOV—deoxyvasicinone (1000 μM).

Melanin is synthesized in the melanosomes of melanocytes, which is transferred to neighboring keratinocytes through dendrites [17,18]. The anti-melanogenic effects of deoxyvasicinone treatment were assessed in the co-culture of MNT-1 and HaCaT, human keratinocyte cell lines. The cells were treated with 1000 μM deoxyvasicinone and 200 nM α-MSH for 24 h, then subjected to *L*-DOPA or FM staining to visualize the distribution of melanosomes (Figure 4A,B). The deoxyvasicinone treatment decreased the number of melanin particles and also attenuated the extension of dendrites, confirming that deoxyvasicinone can attenuate melanocyte activation.

### 2.5. The Effects of Deoxyvasicinone upon Tyrosinase Enzymatic Activity

**Figure 5:**
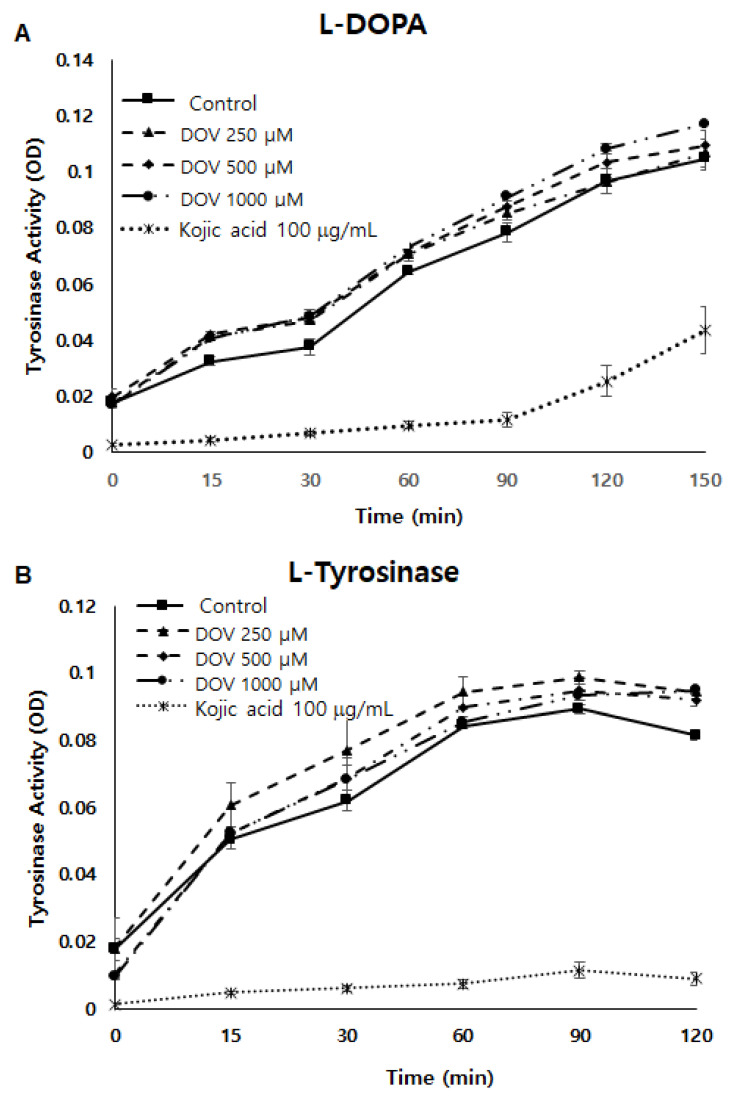

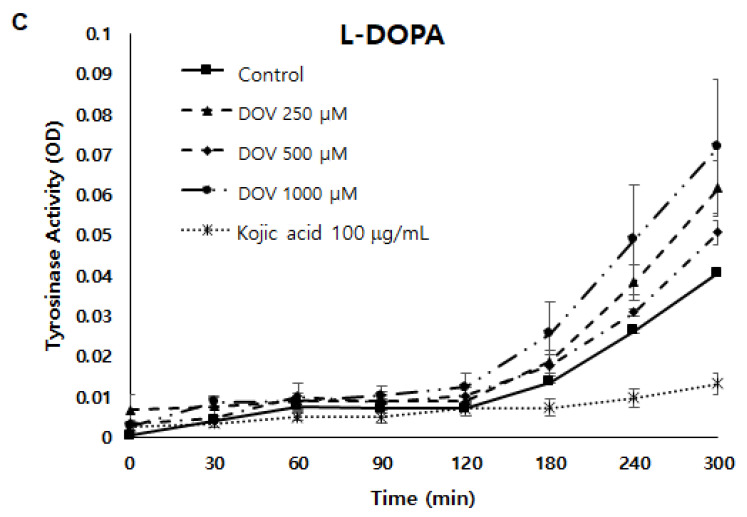
(**A**,**B**) The effect of deoxyvasicinone on tyrosinase enzymatic activity was measured using the cell-free mushroom tyrosinase assay with (**A**) *L*-DOPA and (**B**) *L*-tyrosine as substrates. (**C**) The effects of deoxyvasicinone upon cellular tyrosinase activity of MNT-1 cells was measured using *L*-DOPA as the substrate. Data values are presented as the mean ± SD (*n* = 3).

Because tyrosinase (TYR) plays a major role in the synthesis of melanin [8], a cell-free mushroom TYR assay was first performed to examine whether deoxyvasicinone could directly inhibit TYR enzymatic activity. As shown in Figure 5A,B, TYR enzymatic activity was not inhibited by deoxyvasicinone treatment when using L-DOPA or L-tyrosinase as substrates. Moreover, even when the TYR assay was performed using cellular TYR from MNT-1 human melanoma cells (Figure 5C), deoxyvasicinone was unable to inhibit the enzymatic activity of cellular TYR, demonstrating that deoxyvasicinone did not directly inhibit TYR enzymatic activity. TYR activity was suppressed significantly by kojic acid, which is a positive control.

### 2.6. The Effects of Deoxyvasicinone on mRNA Expression of Melanogenic Enzymes

**Figure 6:**
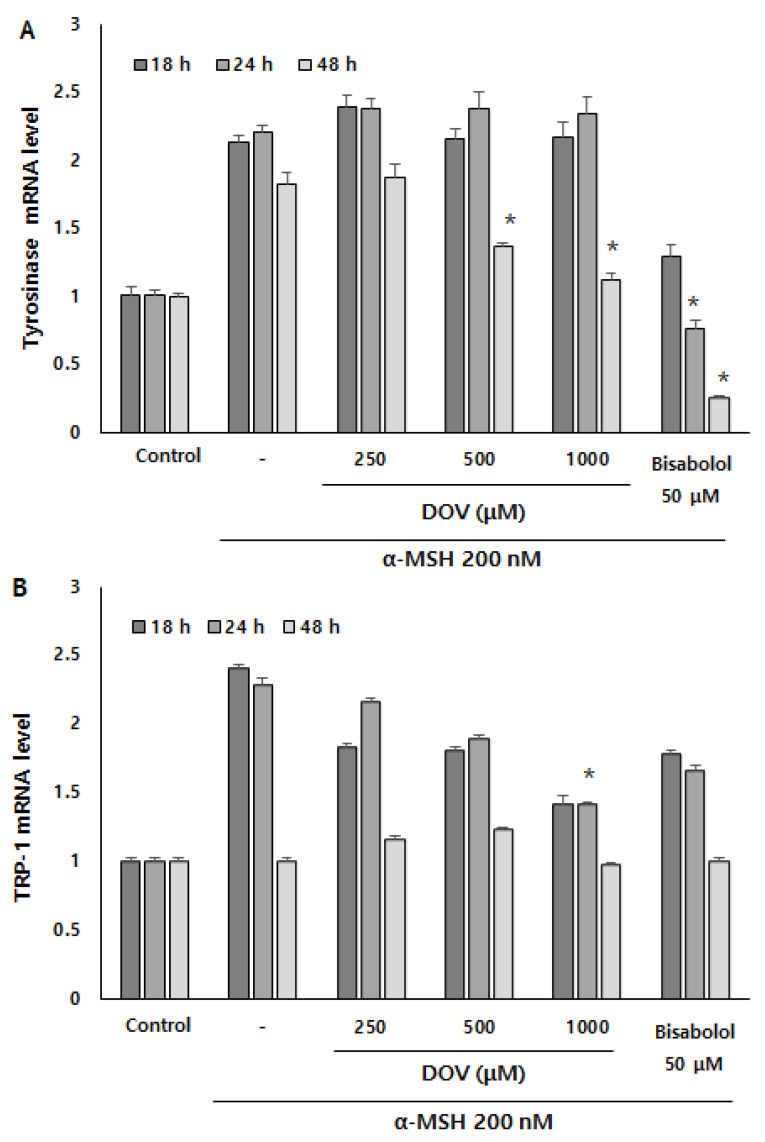

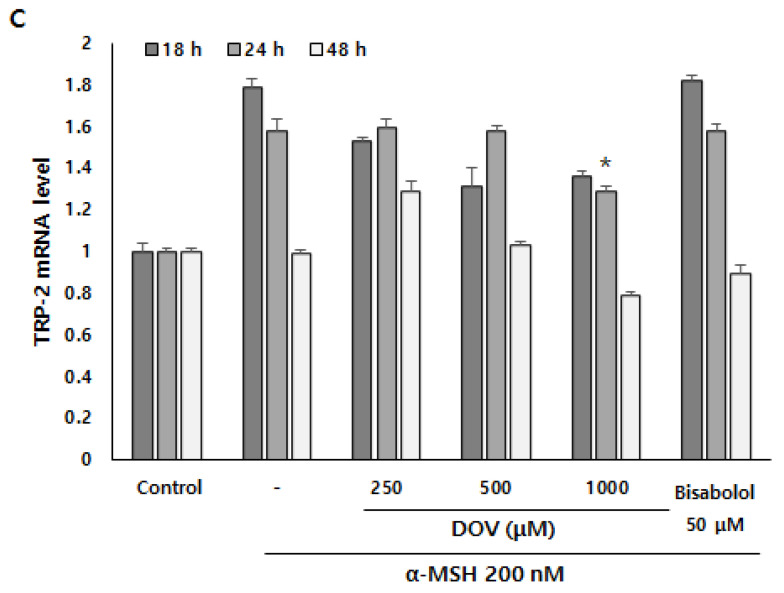
The (**A**) TYR, (**B**) TRP-1, and (**C**) TRP-2 mRNA expression levels in B16F10 cells were measured by real-time PCR upon treatment with α-MSH and various concentrations of deoxyvasicinone or α-bisabolol for 18, 24, or 48 h. Data are presented as the mean ± SD (*n* = 3, * *p* < 0.05). Control—cells treated with neither α-MSH nor chemicals; or DOV—deoxyvasicinone.

Even though the enzymatic activities of melanogenic enzymes were not affected, melanogenesis can also be attenuated by the downregulation of melanogenic enzymes, which results in the reduction of enzyme levels per se [16]. To investigate whether deoxyvasicinone can affect the transcription of melanogenic enzymes, we measured mRNA expression of the melanogenic enzymes TYR, tyrosinase-related protein 1 (TRP-1), and tyrosinase-related protein 2 (TRP-2) using real-time PCR at 18, 24 and 48 h (Figure 6A–C). α-MSH stimulation resulted in the upregulation of TYR, TRP-1, and TRP-2 in B16F10 cells, but deoxyvasicinone treatment significantly suppressed the mRNA expression of TYR at 48 h, and TRP-1 and TRP-2 at 24 h when compared with α-MSH-stimulated cells without deoxyvasicinone. α-bisabolol, a positive control, significantly inhibited the upregulation of TYR only, compared to the α-MSH stimulated group.

### 2.7. The Effects of Deoxyvasicinone on the Artificial Human Pigmented Epidermis Model Melanoderm^TM^

**Figure 7:**
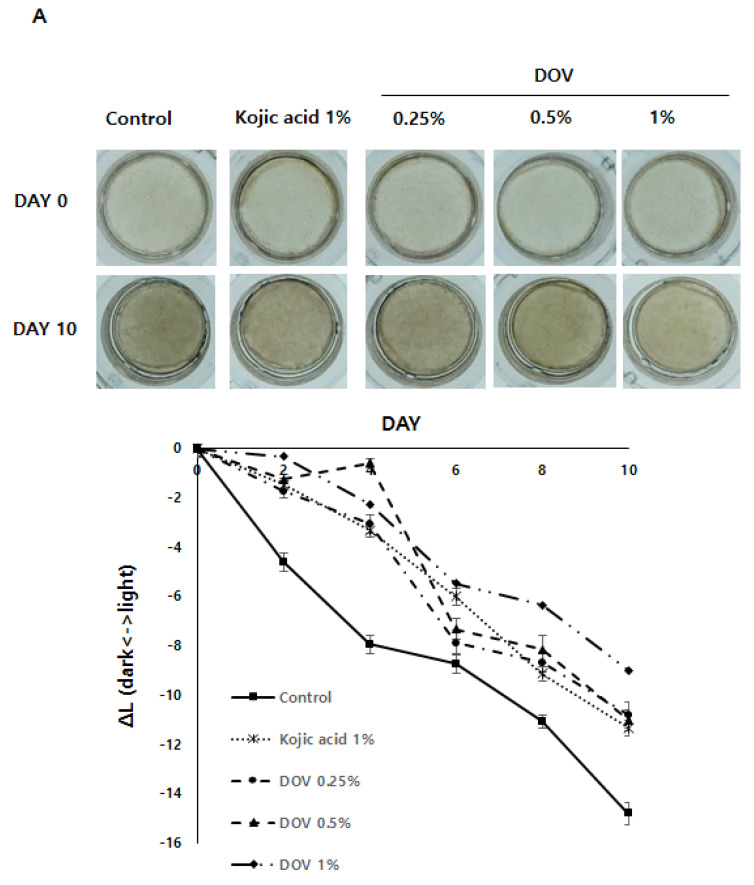

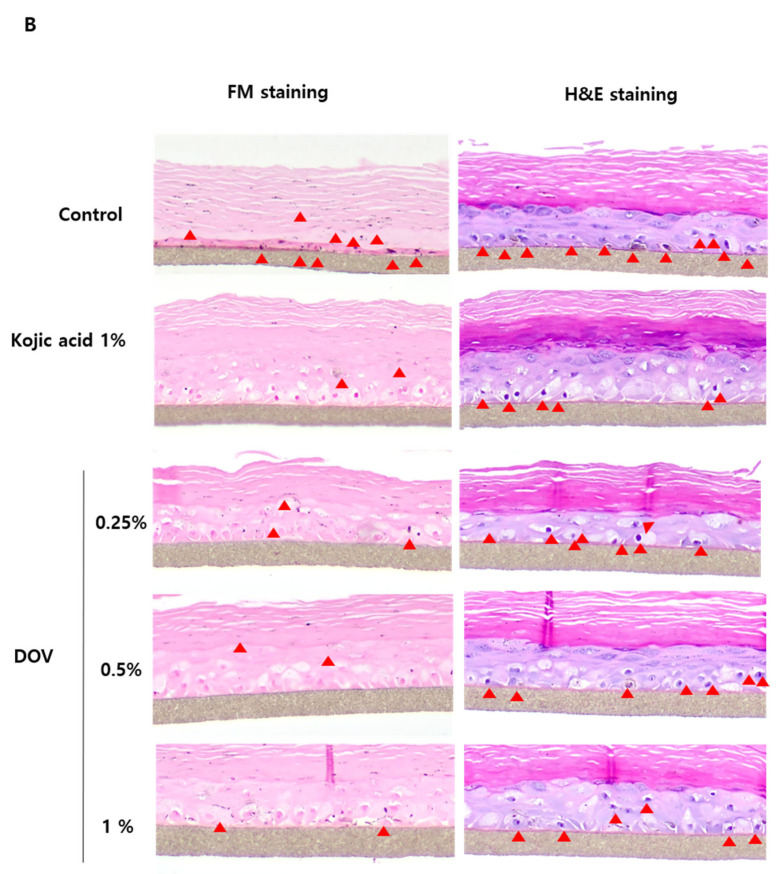
The effects of the deoxyvasicinone treatment on the 3D human pigmented epidermis model Melanoderm^TM^: Melanoderm™ was treated with deoxyvasicinone or kojic acid (1%) every other day for 10 days and (**A**) the color and degree of brightness of the tissues were measured over 10 days using ΔL value of the photographs of the tissues compared with that of Day 0. (**B**) The histology of the tissues treated for 10 days. Left (FM staining) and right (H&E staining). The treatment of deoxyvasicinone or kojic acid led to the attenuation of melanocyte activation and distribution compared with untreated control. Values are presented as the mean ± SD (*n* = 3).

For this investigation, the artificial human pigmented epidermis model Melanoderm^TM^, (MatTek, Ashland, MA, USA), which was reconstructed with normal human keratinocytes and normal melanocytes, was used. Before use, Melanoderm™ was stabilized in a 37 °C CO_2_ incubator for a day and then treated with various concentrations of deoxyvasicinone or 1% kojic acid every other day for 10 days. Tissues were photographed at every treatment, and the ΔL value (changes in the brightness of tissues from Day 0) was analyzed. As shown in Figure 7A,B, untreated control tissues became darker at Day 10 when compared with Day 0, resulting in the minus ΔL value, but deoxyvasicinone treatment attenuated it as confirmed by the brighter tone of the deoxyvasicinone-treated tissues at Day 10 compared with untreated control. The tissues fixed and stained with hematoxylin and eosin (H&E) or FM after 10 days of treatment showed that deoxyvasicinone attenuated melanosome accumulation and dendrite formation of melanocytes in the basal layer of Melanoderm™, which was comparable to the tissue treated with kojic acid (1%).

## 3. Discussion

New anti-melanogenic agents are in high demand with the rapid growth in the global sales of skin-lightening cosmetics [19]. In 2017, the global skin-lightening market value amounted to around USD 4.8 billion [20]. The discovery of natural anti-melanogenic agents is needed in order to develop more effective skin-lightening cosmetics with less adverse effects and nature-friendly images. In this study, we isolated deoxyvasicinone from a marine-derived *Streptomyces* sp. CNQ-617. The IUPAC name for deoxyvasicinone is 2,3-dihydro-1*H*-pyrrolo[2,1-b] quinazolin-9-one. In previous studies, it was suggested that deoxyvasicinone possessed several therapeutic activities, including anti-inflammatory and anti-cancer effects [15]. These activities are commonly associated with quinazoline derivatives [15,21,22]. In addition, synthetic quinazoline derivatives are known to have antioxidant and anti- melanogenesis effects [23,24]. Therefore, we hypothesized that deoxyvasicinone, which is a quinazoline derivative, might have anti-melanogenic effects.

In this study, deoxyvasicinone derived from *Streptomyces* sp. CNQ-617 was shown to inhibit melanin synthesis in murine B16F10 melanoma cells and human MNT-1 melanoma cells. The melanin and cell viability assays demonstrated that the melanin contents of both types of cells were reduced by treatment with 250–1000 µM deoxyvasicinone, with a cell viability retained at greater than 80%. The potency of deoxyvasicinone was not as high as arbutin; however, it reduced melanin content significantly and had minimal cytotoxicity, even at high concentrations.

Deoxyvasicinone was not effective in suppressing enzymatic activities of TYR but appeared to downregulate the expression of melanogenic enzymes. This indicates that deoxyvasicinone may affect the intracellular signaling orchestrating the expression of melanogenic enzymes. The major transcription factor that directly regulates the transcription of TYR, TRP1, and TRP2 is the microphthalmia-associated transcription factor (MITF) [25]. MITF provides central links between transcription factors and signaling pathways in melanocytes for cell survival, proliferation, and differentiation [26], suggesting that deoxyvasicinone may generally affect melanocyte activation. Indeed, deoxyvasicinone was effective in the reduction of melanosome accumulation and dendrite extension in the co-culture of MNT-1 and HaCaT cells, an in vitro system reflecting the cross-talk between melanocytes and keratinocytes [27]. When melanin is synthesized in melanocytes, melanosomes are transferred from melanocyte to keratinocyte by extension of the melanocyte dendrites, and α-MSH stimulation promotes the extension of dendrites through upregulating melanosome transporter proteins [16,28]. The microscopic images of *L*-DOPA-stained co-cultured HaCaT human keratinocytes and MNT-1 cells revealed that the treatment of α-MSH-stimulated groups with deoxyvasicinone caused regression of the cell dendrites. These results appear to support the conclusion that deoxyvasicinone inhibited the activation of melanocytes. Indeed, many bacterial metabolites are known to display anti-melanogenic effects through affecting the expression of melanogenic enzymes [29,30], suggesting that this may be a common target for natural anti-melanogenic agents.

Most importantly, the repeated treatment of deoxyvasicinone effectively suppressed the pigmentation of the artificial 3D pigmented human skin model Melanoderm™, the potency of which was comparable to kojic acid. Considering that kojic acid is a potent TYR inhibitor while deoxyvasicinone failed to inhibit TYR activity, this was a surprising result. We consider that the general suppression of melanocyte activation signaling pathway by deoxyvasicinone, even with low potency, may have resulted in the effective whitening effects after repeated treatment. Furthermore, deoxyvascicinone did not significantly affect the integrity of tissues when compared with 1% kojic acid, suggesting that deoxyvasicinone may be safe to use in humans. The 3D skin model has a viable skin barrier function that is absent in 2D cultured cell models [31]. Therefore, it is regarded as closer to human skin, supporting the idea that deoxyvasicinone can be used as an active anti-melanogenic agent for cosmetic uses on the human skin.

## 4. Materials and Methods

### 4.1. General Experimental

The UV spectra were recorded in MeOH using a Chirascan Plus spectrometer (Applied Photophysics, Randalls Rd, Leatherhead, UK). Low-resolution LC/MS measurements were performed on the Agilent Technologies 1260 quadrupole and Waters Micromass ZQ LC/MS system using a reversed-phase column (Phenomenex Luna C18 (2) 100 Å, 50 mm × 4.6 mm, 5 µm) at a flow rate of 1.0 mL/min at the National Research Facilities and Equipment Center (NanoBioEnergy Materials Center) at Ewha Womans University. NMR spectra were obtained using an Agilent NMR spectrometer (Agilent, Santa Clara, CA, USA, at 400 MHz for ^1^H and at 100 MHz for ^13^C) equipped at the Drug Development Research Core Center (Ewha Womans University) using the signals of the residual solvent as internal references (*δ*_H_ 4.87 and 3.31 ppm, and *δ*_C_ 49.1 ppm for deuterated methanol (CD_3_OD)). Open column chromatography was performed using silica (40–63 μm, Merck silica gel 60, Kenilworth, NJ, USA) eluting with a gradient solvent of dichloromethane (CH_2_Cl_2_) and methanol (MeOH).

### 4.2. Bacterial Strain

The marine actinomycete strain CNQ-617 was isolated from a marine sediment sample collected offshore of La Jolla, CA, USA. The strain was designated as the MAR3 clade on the basis of 16S rDNA analysis. The phylogenetic analysis revealed that this strain showed 99.7% similarity to *Streptomyces cacaoi* according to the results of NCBI blast analysis of the partial 16S rDNA. The gene sequence data are available from Genebank (deposit # EU161093).

### 4.3. Cultivation and Culture Extraction

*Streptomyces* strain CNQ-617 was cultured in 160 of 2.5-L Ultra Yield Flasks, each containing 1 L of the medium (10 g/L of soluble starch, 2 g/L of yeast, 4 g/L of peptone, 10 g/L of CaCO_3_, 20 g/L of KBr, and 8 g/L Fe_2_(SO_4_)_3_·4H_2_O dissolved in 750 mL natural seawater and 250 mL distilled water) at 25 °C with constant shaking at 120 rpm. After 15 days, the broth was extracted with ethyl acetate (EtOAc; 160 L in total) to afford 16.0 g of EtOAc extract.

### 4.4. Isolation

The crude extract (16.0 g) from the CNQ-617 strain was fractionated by medium-pressure liquid chromatography (MPLC) eluting with a step gradient of dichloromethane and methanol (100/0, 99/1, 98/2, 96/4, 95/5, 90/10, 80/20, 50/50, 0/100, *v/v*; 600 mL for each gradient) to obtain fractions M1–M9. Fraction M3 was purified by HPLC (Phenomenex Luna C18(2) 100 Å, 250 mm × 10 mm) with 17% acetonitrile in H_2_O at flow rate 2.0 mL/min to yield 42.1 mg deoxyvasicinone (**1**) (t_R_ 11.0 min).

*Deoxyvasicinone* (**1**): white, amorphous solid; UV (MeOH) λ_max_ (log ε) 207 (4.06), 228 (4.20), 264 (3.63), 300 (3.50), 305 (3.23) nm; ^1^H (400 MHz, CD_3_OD): *δ*_H_ 8.20 (1H, d, *J* = 8.0 Hz), 7.81 (1H, dd, *J* = 8.0, 8.0 Hz), 7.62 (1H, d, *J* = 8.0 Hz), 7.51 (1H, dd, *J* = 8.0, 8.0 Hz), 4.20 (2H, t, *J* = 1.2 Hz, H-9), 3.20 (2H, t, *J* = 1.2 Hz, H-7), 2.31 (2H, m, H-8); ^13^C NMR (100 MHz, CD_3_OD): *δ*_C_ 162.6 (qC, C-10), 162.4 (qC, C-6), 150.1 (qC, C-5), 135.6 (CH, C-3), 127.5 (qC, C-2), 127.2 (CH, C-4), 127.1 (CH, C-1), 121.3 (qC, C-11), 48.0 (CH_2_, C-9), 33.1 (CH_2_, C-7), 20.3 (CH_2_, C-8); LRMS *m*/*z* 187.5 [M+H]^+^.

### 4.5. Cell Culture

The B16F10 cells and HaCaT cells purchased from ATCC were cultured in Dulbecco’s modified Eagle’s medium (DMEM) high-glucose with 10% fetal bovine serum (FBS) and 1% penicillin–streptomycin supplementation. MNT-1 cells were cultured in minimum essential medium with 10% DMEM, 20% FBS, 1M HEPES, and 1% penicillin–streptomycin. All cells were cultured in CO_2_ incubators at 37 °C under a humidified atmosphere of 5% CO_2_. When the cell confluency reached 80%, the cells were sub-cultured using 0.05% trypsin.

### 4.6. Melanin Assay and Cell Viability Assay (MTT)

The B16F10 cells and MNT-1 cells were seeded and cultured for 24 h, then treated for 48 h with various concentrations of deoxyvasicinone, with 250 μM arbutin, or with 0.5% dimethyl sulfoxide (DMSO), and with 200 nM α-MSH in the absence of phenol red. After treatment for 48 h, the melanin content was assessed by measuring the absorbance at 405 nm of cell-dissolved solution with 1 N NaOH using an ELISA reader. To measure the cell viabilities, we treated the cells with 0.5 mg/mL 3-(4,5-dimethylthaizaol-2-yl)-2,5-diphenyltetrazolium bromide (MTT) solution as previously described [32]. After 2 h of incubation, resultant formazan was dissolved with DMSO, and the absorption value of the supernatant was measured at 540 nm using an ELISA reader.

### 4.7. Mushroom Tyrosinase Inhibition Assay

The cell-free mushroom tyrosinase assay was conducted to determine whether deoxyvasicinone directly inhibited tyrosinase enzymatic activity. A total of two hundred fifty (250) units of mushroom tyrosinase were mixed with 180 μL of 0.03% tyrosine in 0.1 M potassium phosphate, and 50 units of mushroom tyrosinase were added to 0.2% L-DOPA in 0.1 M potassium phosphate. Then, 2 μL of deoxyvasicinone or DMSO, or 0.01% kojic acid, was added and mixed at 37 °C using a thermomixer at 350 rpm for the indicated time. The absorbance of the solution was determined at 475 nm.

### 4.8. RNA Isolation and Real-Time PCR

B16F10 cells were seeded at a density of 2 × 10^5^ cells per well. The next day, the cells were treated with deoxyvasicinone and α-MSH and then cultured for 18, 24, and 48 h. The cells were next lysed with TRIzol reagent, and chloroform was added. The aqueous phase of the solution was separated by centrifugation and mixed with isopropanol. After centrifugation, the RNA pallets were gathered and dissolved with RNase-free, diethylpyrocarbonate (DEPC)-treated water.

The relative expression levels of mRNA were then determined via quantitative real-time PCR (qRT-PCR). Using 1250 ng of total RNA and oligo(dT), we synthesized the cDNA. SYBR Green PCR master mix was added, and a StepOnePlusTM Real-time PCR machine was used. The primers sequences of each gene were as follows: forward tyrosinase, 5′-GGG CCC AAA TTG TAC AGA GA-3′; reverse tyrosinase, 5′-ATG GGT GTT GAC CCA TTG TT-3′; forward TRP-1, 5′-GTT CAA TGG CCA GGT CAG CA-3′; reverse TRP-1, 5′-CAG ACA AGA AGC AAC CCC GA-3′; forward TRP-2, 5′-TCC AGA AGT TTG ACA GCC C-3′; reverse TRP-2, 5′-GGA AGG AGT GAG CCA AGT TAT G-3′. The annealing temperature of the experiment was 50 °C.

### 4.9. Skin Whitening Assay Using the Artificial Human Epidermal 3D Skin Model, Melanoderm^TM^

Melanoderm^TM^ (MaTek, Ashland, MA, USA) is a pigmented human epidermis skin model that consists of normal human-derived epidermal keratinocytes and normal human-derived melanocytes [17]. The Melanoderm^TM^ skin was pre-incubated for 24 h, then treated with deoxyvasicinone or 1% kojic acid every other day for 10 days. Tissues were photographed at every treatment and measured of the ΔL values. After 10 days, the samples were fixed by phosphate-buffered formalin and visualized by Fontana–Masson (FM) and hematoxylin and eosin (H&E) staining.

### 4.10. The Statistical Analysis

The data were expressed as the mean ± standard deviation (SD) of three or more independent experiments. The statistical analysis was performed with a two-sided Student’s *t*-test. When the *p*-value of the data was less than 0.05, the data were regarded as significant.

## 5. Conclusions

Herein, a compound from culture broth extract of *Streptomyces* sp. CNQ-617, namely, deoxyvasicinone (**1**), was shown to effectively modulate melanogenesis by downregulating the expression of melanogenic enzymes. Although the efficacy of deoxyvasicinone was weaker than that of arbutin, the repeated treatment of deoxyvasicinone manifested whitening effects in the 3D pigmented human epidermis model without significant toxicity, suggesting that it can be used safely as a novel anti-melanogenic agent in humans.

## Data Availability

The data presented in this study are available on request from the corresponding author.

## Supplementary Materials

The following supporting information can be downloaded at: https://www.mdpi.com/article/10.3390/md20020155/s1, Figure S1. ^1^H NMR spectrum (400 MHz, CD_3_OD) of doexyvasicinone (**1**); Figure S2. ^13^C NMR spectrum (100 MHz, CD_3_OD) of doexyvasicinone (**1**).
