# Supplementary material for: Deoxyvasicinone with Anti-Melanogenic Activity from Marine-Derived Streptomyces sp. CNQ-617
Source: Mar Drugs. 2022 Feb 21;20(2):155. doi: 10.3390/md20020155 (PMC8874703; doi:10.3390/md20020155)

Supporting Information for

Deoxyvasicinone with the anti-melanogenic activity from a marine-derived *Streptomyces* sp. CNQ-617

Se-eun Lee[1,†], Min-ju Kim[2,†], Prima F. Hillman[3], Dong-Chan Oh[4], William Fenical[5], Sang-Jip Nam[3,*], and Kyung-Min Lim[1,*]

1 College of Pharmacy, Ewha Womans University, Seoul 03760, Korea; 0213lse@naver.com (S.L.), kmlim@ewha.ac.kr (K.-M.L.)

2 The Graduate School of Industrial Pharmaceutical Sciences, Ewha Womans University, Seoul 03760, Republic of Korea; bm4976@gmail.com (M.K.)

3 Department of Chemistry and Nanoscience, Ewha Womans University, Seoul, 03760, Republic of Korea; primafitriah@gmail.com (P.F.H.), sjnam@ewha.ac.kr (S.-J.N.)

4 Natural Products Research Institute, College of Pharmacy, Seoul National University, Seoul 08826, Korea; dongchanoh@snu.ac.kr (D.-C.O.)

5 Center for Marine Biotechnology and Biomedicine, Scripps Institution of Oceanography, University of California-San Diego, CA 92093-0204, USA; wfenical@ucsd.edu (W.F.)

* Correspondence: sjnam@ewha.ac.kr (S.-J.N.), kmlim@ewha.ac.kr (K.-M.L.)

† These authors contributed equally to this work

Table of Contents

Figure S1. ^{1}H NMR spectrum (400 MHz, CD_3OD) of deoxyvasicinone (**1**) ········ **S3**

Figure S2. ^{13}C NMR spectrum (100 MHz, CD_3OD) of deoxyvasicinone (**1**) ········ **S4**

Figure S1. ^{1}H NMR spectrum (400 MHz, CD_3OD) of doexyvasicinone (**1**)

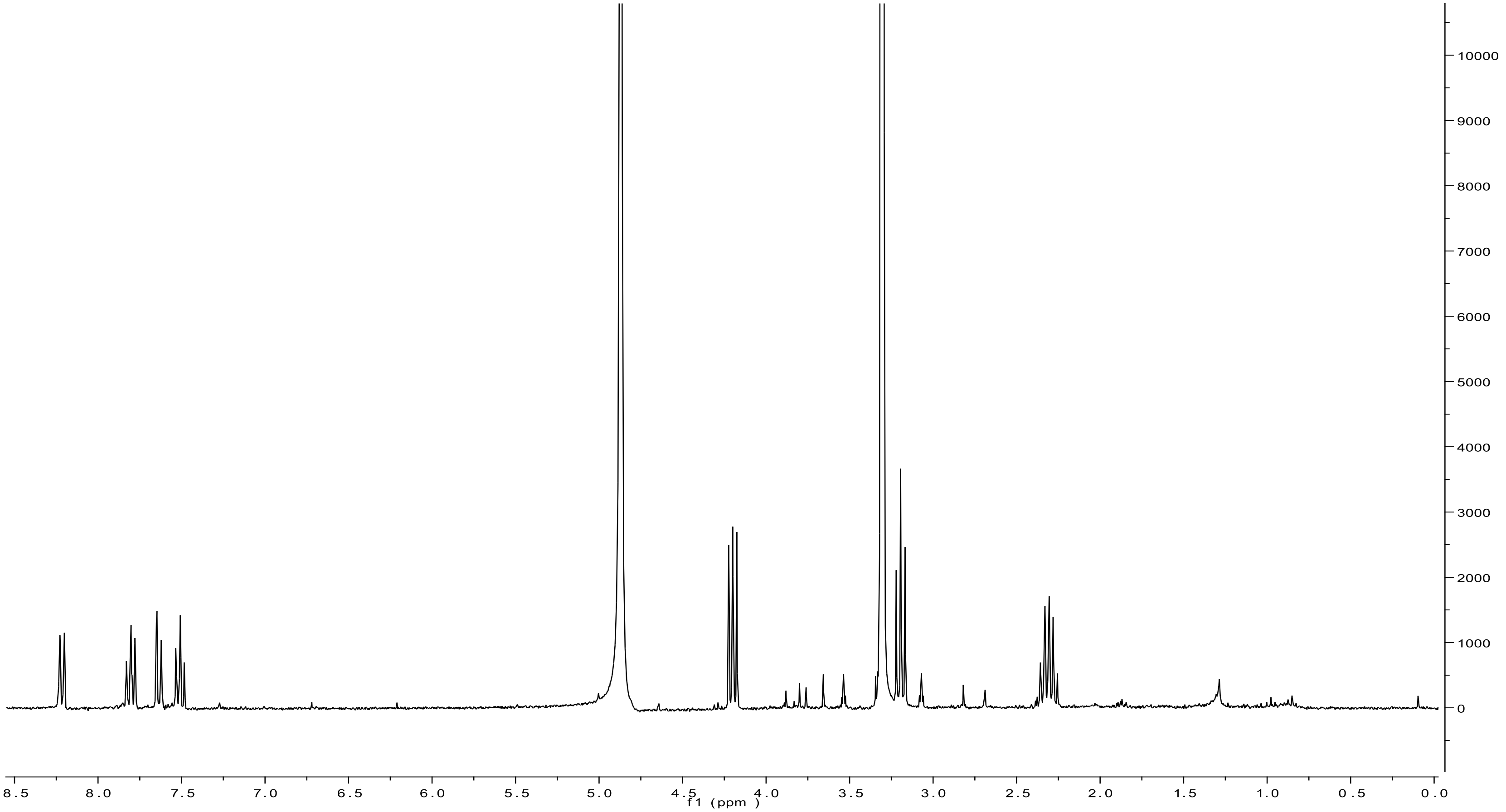

Figure S2. ^{13}C NMR spectrum (100 MHz, CD_3OD) of doexyvasicinone (**1**)

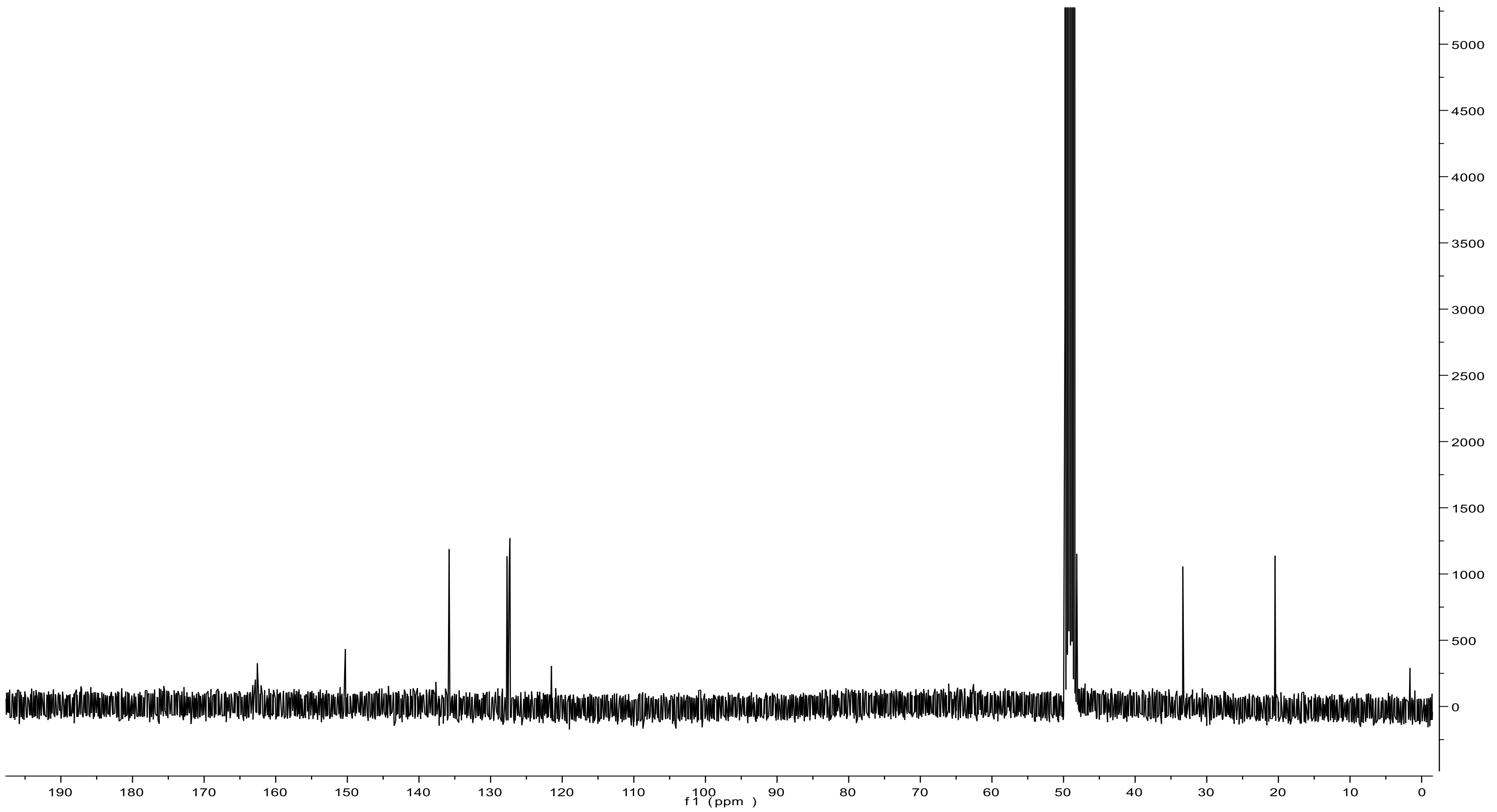

Supplement: Supplementary file 1 [file marinedrugs-20-00155-s001.zip › marinedrugs-1592020-supplementary.pdf]
